# Coexisting Hyperuricemia and High Urinary Uric Acid Excretion Are Associated With a Higher Prevalence of Obesity and Metabolic Syndrome in Type 2 Diabetes

**DOI:** 10.1155/jdr/8461568

**Published:** 2026-10-26

**Authors:** Man-Rong Xu, Meng-Han Li, Cui-Chun Zhao, Yu-Jie Wang, Ming-Yun Chen, Ya-Wen Zhang, Jun-Xi Lu, Chun Yue, Lu-Lu Xu, Zhi-Feng Sheng, Lian-Xi Li

**Affiliations:** ^1^ Department of Endocrinology and Metabolism Shanghai Sixth People′s Hospital Affiliated to Shanghai Jiao Tong University School of Medicine Shanghai Clinical Center for Diabetes Shanghai Diabetes Institute Shanghai Key Laboratory of Diabetes Mellitus Shanghai Key Clinical Center for Metabolic Disease Shanghai China sjtu.edu.cn; ^2^ Department of Endocrinology The First Affiliated Hospital of Fujian Medical University Fuzhou Fujian China fjmu.edu.cn; ^3^ Health Management Center National Clinical Research Center for Metabolic Diseases Hunan Provincial Clinical Medicine Research Center for Intelligent Management of Chronic Disease Hunan Provincial Key Laboratory of Metabolic Bone Diseases Department of Metabolism and Endocrinology The Second Xiangya Hospital of Central South University Changsha Hunan China csu.edu.cn

**Keywords:** abdominal obesity, metabolic syndrome, obesity, serum uric acid, Type 2 diabetes, urine uric acid excretion

## Abstract

**Purpose:**

Both hyperuricemia and high urinary uric acid excretion (UUAE) are strongly associated with obesity and metabolic syndrome (MetS). However, few studies have examined the joint association of these two conditions with obesity and MetS. This study was aimed at investigating whether the concomitant presence of high serum uric acid (SUA) and high UUAE was associated with higher odds of obesity and MetS in patients with Type 2 diabetes mellitus (T2DM).

**Methods:**

This cross‐sectional study included 7051 Chinese patients with T2DM in a real‐world setting. Participants were divided into four groups, including patients with normal SUA and UUAE (Group 1), normal SUA and high UUAE (Group 2), high SUA and normal UUAE (Group 3), and high SUA and UUAE (Group 4). The clinical characteristics and prevalence of obesity and MetS were compared among these groups.

**Results:**

After adjustment, compared with Group 1, both Groups 2 and 3 had a significantly elevated prevalence of obesity (42.6%, 63.2%, and 62.6%, respectively, *p* < 0.001 for trend), abdominal obesity (62.4%, 76.5%, and 81.3%, respectively, *p* < 0.001 for trend), and MetS (66.4%, 78.3%, and 87.8%, respectively, *p* < 0.001 for trend). Moreover, the prevalence of obesity, abdominal obesity, and MetS further increased in Group 4 (84.6% for obesity, 93.6% for abdominal obesity, and 88.5% for MetS). Additionally, serum CRP levels and HOMA2‐IR values were significantly higher in the other three groups than in Group 1, with the highest values in Group 4 (*p* < 0.001 for trend).

**Conclusion:**

The concomitant presence of elevated SUA and UUAE was associated with higher odds of both obesity and MetS among patients with T2DM. Thus, the combined consideration of SUA and UUAE levels may help to identify those T2DM patients with a greater burden of obesity and MetS.

## 1. Introduction

The prevalence of obesity and abdominal obesity has been steadily increasing worldwide in recent decades. Based on the 2015–2019 national survey of obesity prevalence, a recent study in China estimated that the prevalence of overweight in adults was 34.3%, whereas the prevalence of obesity was 16.4% [1]. In addition, a study of over 70,000 subjects revealed a significant increase in abdominal obesity prevalence, rising from 20.2% in 1993 to 46.9% in 2015 [2]. Furthermore, metabolic syndrome (MetS) is defined as a clustering of abdominal obesity, elevated blood pressure, hyperglycemia, hypertriglyceridemia, and decreased high‐density lipoprotein cholesterol (HDL‐C) [3]. Its global burden has grown rapidly, with recent meta‐analysis data showing that the worldwide prevalence ranges from 12.5% to 31.4% [4].

Obesity has been shown to be strongly associated with an increased risk of major noncommunicable diseases, such as nonalcoholic fatty liver disease (NAFLD) [5], cardiovascular disease, different types of cancer, and premature death [6]. A recent study found that patients with Type 2 diabetes mellitus (T2DM) who had metabolic diseases such as obesity were at higher risk of macrovascular and microvascular complications compared to those without [7]. Furthermore, a prospective study of over 50,000 people found that independent of the effect of body mass index (BMI), the risk of NAFLD was obviously higher in participants with abdominal obesity (HR 1.31, 95% CI: 1.26–1.37) [5]. Moreover, Sluik et al. observed that abdominal obesity has a greater contribution to mortality than general obesity in patients with diabetes [8]. As a clustered manifestation of metabolic dysfunction, MetS confers particularly elevated health risks, with a recent cohort study demonstrating increased risks of cardiovascular disease and mortality among individuals with MetS [9].

Uric acid (UA), a recognized end product of purine catabolism, is produced by the action of xanthine oxidase (XO) [10]. Interestingly, UA exerts a dual role as an extracellular antioxidant and as an intracellular promoter of oxidation [11], which may depend on the complex cellular microenvironment [10]. In clinical practice, serum uric acid (SUA) has been confirmed to be correlated with metabolic‐related diseases such as diabetes mellitus [12], metabolic‐associated fatty liver disease (MAFLD) [13], and atherosclerosis [14]. Previous studies also suggested a positive association of increased SUA levels with hypertension (HTN) and MetS [15], chronic kidney disease (CKD) and albuminuria, and the incidence and progression of obesity in T2DM [16, 17]. Moreover, in a cohort study with more than 5 years of follow‐up, a significantly positive association was observed between high levels of SUA and obesity‐related indicators such as BMI and waist circumference (WC), as well as the occurrence of MetS [18].

However, compared with the research on SUA, few investigations have been conducted to explore the relationship between urinary uric acid excretion (UUAE) and metabolic diseases, especially in diabetic populations. A recent study conducted by the present research group found a strong association of UUAE with obesity and abdominal obesity in T2DM patients without CKD [19]. Similarly, Li et al. reported a progressive elevation in BMI across UUAE tertiles in CKD patients [20]. However, the assessment of whether the coexistence of high SUA and UUAE is associated with higher odds of obesity and MetS compared to only elevated SUA or UUAE has not been performed so far.

Therefore, the aim of the present study was to investigate the relationship of SUA and UUAE with obesity, abdominal obesity, and MetS and to identify whether their coexistence was associated with even higher odds of these metabolic diseases in T2DM. To date, it is the first study to evaluate the associations of combined high SUA and UUAE with obesity and MetS in T2DM.

## 2. Methods

### 2.1. Study Design and Population

This cross‐sectional study was conducted in a real‐world clinical setting. The present study was approved by the Ethics Committee of Shanghai Sixth People′s Hospital affiliated to Shanghai Jiao Tong University School of Medicine (Approval Number: 2018‐KY‐018(K)). Prior to participation in this study, all subjects provided written informed consent. Patients with baseline T2DM were consecutively recruited from the Department of Endocrinology and Metabolism, Shanghai Sixth People′s Hospital affiliated to Shanghai Jiao Tong University School of Medicine. Patients were excluded if they (1) were younger than 18 years of age; (2) had incomplete clinical data; (3) had acute diabetic complications, such as diabetic ketoacidosis; (4) were taking drugs that may interfere with UA metabolism, such as losartan, thiazide diuretics, and allopurinol; (5) had systemic inflammatory disease; and (6) had progressive malignancy. Ultimately, the present study included 7051 subjects.

### 2.2. Exposure and Other Covariates

Urinary UA concentration was measured using an enzymatic method. UUAE was calculated by multiplying this concentration by the total urine volume obtained from a complete 24‐h urine collection and was expressed as micromoles per 24 h. SUA levels were measured from venous blood samples. Based on previous studies conducted by the present research group [15], hyperuricemia was defined as SUA levels ≥ 420 *μ*mol/L in men and ≥ 360 *μ*mol/L in women, respectively. Moreover, the normal physiological range of UUAE in adults has not been precisely defined because of variation in age, sex, dietary purine intake, and measurement‐related factors [21]. Given the lack of a universally standardized adult UUAE reference range, high UUAE was defined as ≥ 4880 *μ*mol/24 h, the upper reference limit used by the hospital laboratory; the use of 24‐h UUAE to stratify patients with T2DM has been described previously by the same research group [17]. Participants were stratified into four groups according to the upper limit of the normal range for SUA and UUAE: Group 1, normal SUA and normal UUAE; Group 2, normal SUA and high UUAE; Group 3, high SUA and normal UUAE; and Group 4, high SUA and high UUAE.

As baseline indicators, serum or plasma biomarkers were measured using biochemical assays in samples collected from each participant after an overnight fast of 8–10 h and 2 h after a meal, including SUA, fasting plasma glucose (FPG), 2‐h postprandial plasma glucose (2‐h PPG), fasting C‐peptide (FCP), 2‐h postprandial C‐peptide (2‐h C‐P), glycosylated hemoglobin A1c (HbA1c), triglycerides (TGs), total cholesterol (TC), HDL‐C, low‐density lipoprotein cholesterol (LDL‐C), alanine aminotransferase (ALT), creatinine (Cr), urinary albumin excretion (UAE), and CRP. The homeostasis model assessment for insulin resistance (HOMA2‐IR) scores were then calculated using HOMA2 Calculator 2.2.3 with FPG and FCP values. Moreover, estimated glomerular filtration rate (eGFR) was calculated using the Chronic Kidney Disease Epidemiology Collaboration (CKD‐EPI) equation [22].

Physical examination indicators included body weight, height, WC, hip circumference (HC), systolic blood pressure (SBP), and diastolic blood pressure (DBP). BMI was calculated as body weight in kilograms divided by the square of height in meters. Waist‐to‐hip ratio (WHR) was calculated as WC divided by HC, and waist‐to‐height ratio (WHtR) was calculated as WC divided by height.

Furthermore, all participants reported their smoking and alcohol intake status and had comprehensive datasets including duration of diabetes (DD), history of HTN, and the use of medications such as sulfonylureas (SUs), metformin, and insulin or insulin analogues (IIAs).

### 2.3. Assessment of Outcomes

As previously reported [16], obesity was defined as a BMI ≥ 25 kg/m^2^ and was further classified as mild obesity (25 ≤ BMI < 30 kg/m^2^), moderate obesity (30 ≤ BMI < 35 kg/m^2^), or severe obesity (BMI ≥ 35 kg/m^2^). Furthermore, abdominal obesity was defined as a WC ≥ 90 cm for men and ≥ 80 cm for women, respectively [16]. Mild abdominal obesity was defined as a WC ≥ 90 and < 100 cm in men and ≥ 80 and < 90 cm in women, moderate abdominal obesity as a WC ≥ 100 and < 110 cm in men and ≥ 90 and < 100 cm in women, and severe abdominal obesity as a WC ≥ 110 cm in men and ≥ 100 cm in women [19].

MetS was defined according to the updated NCEP ATP III criteria for Asian Americans [15]. Considering that T2DM or hyperglycemia is one of the components of MetS, the diagnosis in the present T2DM cohort was determined by the presence of at least two of the following: (1) meeting the criteria for abdominal obesity as described above, (2) TG ≥ 1.7 mmol/L, (3) HDL‐C < 1.03 mmol/L in men or < 1.30 mmol/L in women, and (4) BP ≥ 130/85 mmHg or current use of antihypertensive medication.

### 2.4. Statistical Analyses

Normally distributed continuous variables were expressed as mean ± standard deviation (SD) and non‐normally distributed variables as median (interquartile range [IQR]). Qualitative data were summarized as numbers and percentages. For normally distributed variables, the one‐way analysis of variance (ANOVA) with least significant difference (LSD) was applied to evaluate between‐group differences, whereas the Kruskal–Wallis test was used for comparisons of non‐normally distributed variables. Categorical variables were compared using a chi‐square test. Binary logistic regression was performed to evaluate the differences between groups, both without and with adjustment for confounders. Six models were established for adjustment: Model 1 was unadjusted. Model 2 was adjusted for age, gender, and DD. Model 3 was further adjusted for HTN, smoking, and alcohol consumption. Model 4 was further adjusted for the use of IIAs, metformin, and SUs. Model 5 was additionally adjusted for SBP and DBP. Model 6 was further adjusted for ALT, TC, HDL‐C, LDL‐C, TG, eGFR, Cr, UAE, FPG, 2‐h PPG, HbA1c, FCP, 2‐h C‐P, and CRP. All statistical analyses were conducted using SPSS 15.0 software. *p* < 0.05 was accepted as statistically significant.

## 3. Results

### 3.1. Characteristics of the Study Subjects

Table 1 presents the comparison of clinical characteristics among the four groups. There were significant differences in age and gender across the four groups (*p* < 0.001). After adjustment for age and gender, smoking, alcohol consumption, HTN, the use of metformin and IIAs, SBP, DBP, FPG, 2‐h PPG, HbA1c, FCP, 2‐h C‐P, HOMA2‐IR, TG, TC, HDL‐C, ALT, Cr, UAE, and eGFR presented remarkable differences among the four groups (*p* < 0.05 for all). Among them, FCP, 2‐h C‐P, HOMA2‐IR, TG, and UAE levels showed a clear upward trend across the four groups (*p* < 0.001 for all). Furthermore, DBP, TC, ALT, and the use of metformin demonstrated significantly higher levels in patients with high SUA and/or UUAE than in those with normal SUA and UUAE (*p* < 0.05 for all), whereas HDL‐C levels exhibited an opposite trend (*p* = 0.022).

**Table 1:** Characteristics of study subjects according to four groupings based on the levels of SUA and UUAE.

Variables	Patients with normal SUA and UUAE (*n* = 5327)	Patients with normal SUA and high UUAE (*n* = 400)	Patients with high SUA and normal UUAE (*n* = 1246)	Patients with high SUA and UUAE (*n* = 78)	*p* value	*p* value ^∗^
Male (*n*, %)	3085 (57.9%)	296 (74.0%)	638 (51.2%)	55 (70.5%)	< 0.001	< 0.001
Age (years)	57 ± 12	52 ± 11	59 ± 13	48 ± 16	< 0.001	< 0.001
DD (months)^a^	84 (24–144)	60 (12–120)	96 (36–156)	36 (24–87)	< 0.001	0.170
Smoking (*n*, %)	1729 (32.5%)	198 (49.5%)	324 (26.0%)	28 (35.9%)	< 0.001	< 0.001
Alcohol (*n*, %)	929 (17.4%)	121 (30.3%)	196 (15.7%)	16 (20.5%)	< 0.001	0.007
Hypertension (*n*, %)	2488 (46.7%)	187 (46.8%)	840 (67.4%)	45 (57.7%)	< 0.001	< 0.001
Metformin (*n*, %)	3165 (59.4%)	275 (68.8%)	779 (62.5%)	49 (62.8%)	0.001	0.004
SUs (*n*, %)	3399 (63.8%)	240 (60.0%)	792 (63.6%)	37 (47.4%)	0.012	0.179
IIAs (*n*, %)	3775 (70.9%)	290 (72.5%)	849 (68.1%)	44 (56.4%)	0.008	0.009
SBP (mmHg)	131 ± 17	131 ± 16	136 ± 18	129 ± 15	< 0.001	< 0.001
DBP (mmHg)	80 ± 9	82 ± 10	81 ± 10	82 ± 10	< 0.001	< 0.001
FPG (mmol/L)^a^	7.8 (6.2–9.9)	8.5 (7.2–10.6)	7.5 (6.2–9.3)	8.0 (6.4–11.0)	< 0.001	< 0.001
2‐h PPG (mmol/L)^a^	13.3 (10.1–16.8)	14.4 (11.3–17.3)	12.7 (9.6–16.1)	13.2 (10.1–18.1)	< 0.001	0.003
HbA1c (%)	9.07 ± 2.29	9.31 ± 2.06	8.47 ± 2.13	8.67 ± 2.19	< 0.001	< 0.001
FCP (ng/mL)^a^	1.66 (1.06–2.37)	1.92 (1.25–2.83)	2.34 (1.55–3.18)	2.79 (1.83–3.90)	< 0.001	< 0.001
2‐h C‐P (ng/mL)^a^	3.71 (2.16–5.63)	4.24 (2.53–6.21)	5.08 (3.13–7.00)	5.65 (3.30–7.99)	< 0.001	< 0.001
HOMA2‐IR^a^	1.44 (0.90–2.04)	1.72 (1.13–2.50)	1.95 (1.30–2.65)	2.50 (1.66–3.33)	< 0.001	< 0.001
TG (mmol/L)^a^	1.35 (0.95–1.99)	1.79 (1.20–2.80)	1.87 (1.31–2.70)	2.16 (1.49–3.80)	< 0.001	< 0.001
TC (mmol/L)	4.73 ± 1.16	4.91 ± 1.17	4.93 ± 1.27	4.80 ± 1.18	< 0.001	0.004
HDL‐C (mmol/L)	1.14 ± 0.31	1.03 ± 0.28	1.05 ± 0.29	0.97 ± 0.22	< 0.001	0.022
LDL‐C (mmol/L)	3.04 ± 0.91	3.06 ± 1.01	3.08 ± 1.00	2.83 ± 0.93	0.130	0.121
ALT (U/L)^a^	19 (14–29)	25 (16–40)	22 (15–35)	44 (22–72)	< 0.001	< 0.001
Cr (*μ*mol/L)^a^	65 (54–76)	63 (63–75)	75 (61–95)	71 (59–81)	< 0.001	< 0.001
UAE (mg/24 h)^a^	10 (6–23)	18 (10–43)	18 (8–75)	21 (11–127)	< 0.001	< 0.001
eGFR (mL/min/1.73 m^2^)^a^	115 (97–136)	127 (103–148)	95 (73–116)	112 (94–137)	< 0.001	< 0.001

*Note:* Values are expressed as the mean ± SD or median with interquartile range or percentages.

Abbreviations: 2‐h C‐P, 2‐h postprandial C‐peptide; 2‐h PPG, 2‐h postprandial plasma glucose; ALT, alanine aminotransferase; Cr, creatinine; DD, diabetes duration; eGFR, estimated glomerular filtration rate; FCP, fasting C‐peptide; FPG, fasting plasma glucose; HDL‐C/LDL‐C, high‐density/low‐density lipoprotein cholesterol; HOMA2‐IR, updated homoeostasis model assessment of insulin sensitivity/insulin resistance; HTN, hypertension; IIAs, insulin or insulin analogues; SBP/DBP, systolic/diastolic blood pressure; SUA, serum uric acid; SUs, sulfonylureas; TC, total cholesterol; TGs, total triglycerides; UAE, urinary albumin excretion; UUAE, urine uric acid excretion.

^a^The Kruskal–Wallis test was applied. *p* values not adjusted for age and gender for trend.

^∗^ *p* values adjusted for age and gender for trend.

### 3.2. Comparisons of the Prevalence of Obesity and MetS Stratified by Sex, Age, and DD

Figure 1 compares the prevalence of obesity and MetS stratified by sex, age, and DD in T2DM. There was no significant difference in the prevalence of obesity between men and women after controlling for age and DD (Figure 1A, 48.6% for men and 46.7% for women, *p* = 0.444). However, the prevalence of abdominal obesity and MetS were both considerably higher in women than in men (Figure 1A, 56.6% for men and 81.0% for women in abdominal obesity and 65.0% for men and 79.4% for women in MetS, both *p* < 0.001). Notably, the obesity prevalence showed a significant decreasing trend with increasing age, whereas there was a significant increase in the prevalence of MetS (Figure 1B, *p* < 0.001 for trend in obesity and MetS). However, the changing trend in abdominal obesity was not significant after adjusting for sex and DD (Figure 1B, *p* = 0.515 for trend). In addition, there was an obvious difference in the prevalence of obesity and abdominal obesity among different DD groups, but without an apparent trend (Figure 1C, *p* = 0.006 for trend in obesity and *p* = 0.002 for trend in abdominal obesity). In terms of MetS, the prevalence showed a positive association with increasing DD, but without statistical difference (Figure 1C, *p* = 0.140 for trend).

**Figure 1:**
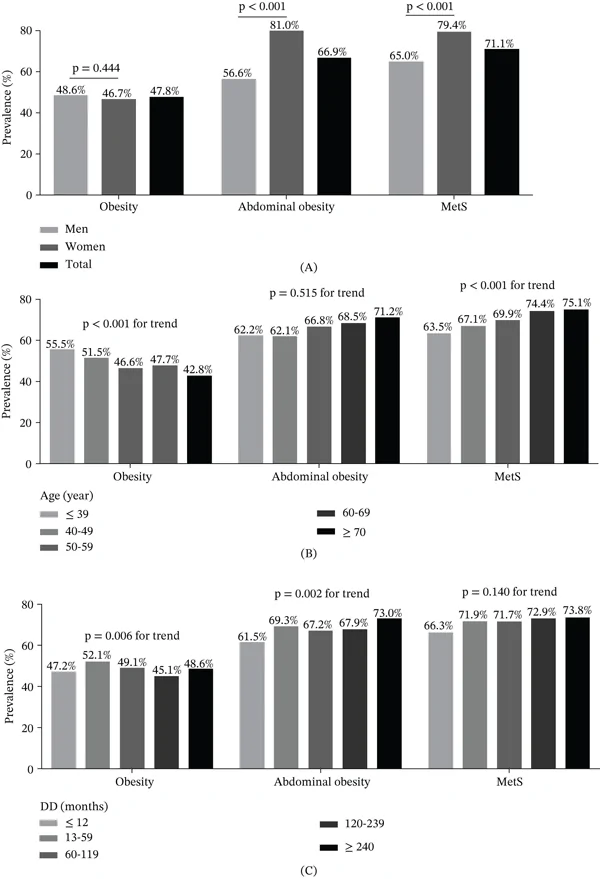
Comparisons of the prevalence of obesity and MetS stratified by sex, age, and DD. (A) Comparison of the prevalence of obesity, abdominal obesity, and MetS between men and women patients with T2DM after adjusting for age and DD (*p* = 0.444 in obesity and *p* < 0.001 in abdominal obesity and MetS). (B) Comparison of the prevalence of obesity, abdominal obesity, and MetS stratified by age after adjusting for sex and DD (*p* < 0.001 for trend in obesity and MetS and *p* = 0.515 for trend in abdominal obesity). (C) Comparison of the prevalence of obesity, abdominal obesity, and MetS stratified by DD after adjusting for age and sex (*p* = 0.006 for trend in obesity, *p* = 0.002 for trend in abdominal obesity, and *p* = 0.140 for trend in MetS).

### 3.3. Comparisons of the Obesity and MetS Prevalence in Different Groups

Figure 2 illustrates that the prevalence of obesity, abdominal obesity, and MetS was significantly higher in Groups 2 and 3 than in Group 1. More importantly, the prevalence of obesity was further increased in Group 4 compared with Groups 2 and 3, almost double that of Group 1 (Figure 2A, 42.6%, 63.2%, 62.6%, and 84.6% for the four groups in obesity, respectively; Figure 2B, 62.4%, 76.5%, 81.3%, and 93.6% for the four groups in abdominal obesity, respectively; and Figure 2C, 66.4%, 78.3%, 87.8%, and 88.5% for the four groups in MetS, respectively, *p* < 0.001 for all trends). Furthermore, the patients in Group 1 had the lowest prevalence of severe obesity compared to Groups 2, 3, and 4, and the highest prevalence was observed in the patients in Group 4 (Figure 2D, 0.5%, 1.8%, 1.1%, and 5.1%, *p* < 0.001 for obesity trends). Similarly, the prevalence of severe abdominal obesity was noticeably higher in Groups 2 and 3 than in Group 1, whereas the prevalence of severe abdominal obesity further increased in Group 4 (Figure 2E, 6.7%, 13.0%, 14.2%, and 20.5%, *p* < 0.001 for abdominal obesity trends). Moreover, patients in Group 4 showed the highest prevalence of combined four or more MetS components (Figure 2F, 26.7%, 34.3%, 35.6%, and 39.7% for four MetS components and 12.2%, 20.5%, 30.3%, and 34.6% for five MetS components, *p* < 0.001 for trend in the number of MetS components).

**Figure 2:**
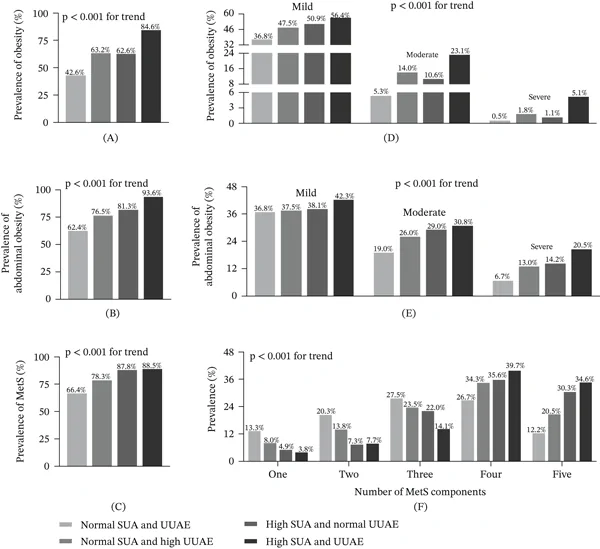
Comparisons of the prevalence of obesity and MetS in different groups. (A) Comparisons of the prevalence of obesity among Group 1, Group 2, Group 3, and Group 4 after adjusting for gender, age, and DD (*p* < 0.001 for trend). (B) Comparisons of the prevalence of abdominal obesity among Group 1, Group 2, Group 3, and Group 4 after adjusting for gender, age, and DD (*p* < 0.001 for trend). (C) Comparisons of the prevalence of MetS among Group 1, Group 2, Group 3, and Group 4 after adjusting for gender, age, and DD (*p* < 0.001 for trend). (D) Comparisons of the prevalence of mild, moderate, and severe obesity separately among Group 1, Group 2, Group 3, and Group 4 after adjusting for gender, age, and DD (*p* < 0.001 for trend). (E) Comparisons of the prevalence of mild, moderate, and severe abdominal obesity separately among Group 1, Group 2, Group 3, and Group 4 after adjusting for gender, age, and DD (*p* < 0.001 for trend). (F) Comparisons of the prevalence of different numbers of MetS components among Group 1, Group 2, Group 3, and Group 4 after adjusting for gender, age, and DD (*p* < 0.001 for trend).

### 3.4. Comparisons of Parameters Related to Obesity and MetS Among the Four Groups

Figure 3 compares obesity‐related parameters (BMI, WC, WHR, and WHtR) and MetS‐related indicators (TG and HDL‐C) among the four groups. After controlling for age, sex, and DD, there were significantly higher BMI, WC, and WHtR in Group 2 and Group 3 compared with Group 1, whereas WHR in Group 2 was the highest among the four groups (Figure 3A–D, *p* < 0.001 for all trends). It is worth noting that the patients in Group 4 exhibited notably higher BMI, WC, and WHtR compared to those in Group 2 and Group 3 (Figure 3A,B,D, *p* < 0.001 for all trends). Similarly, higher levels of TG were observed in Groups 2, 3, and 4 compared with Group 1, with the highest levels observed in Group 4 (Figure 3E, *p* < 0.001 for trend). In contrast, HDL‐C levels were significantly lower in Groups 2 and 3 than in Group 1. HDL‐C level in Group 4 was further decreased compared with Groups 2 and 3 (Figure 3F, *p* < 0.001 for trend).

**Figure 3:**
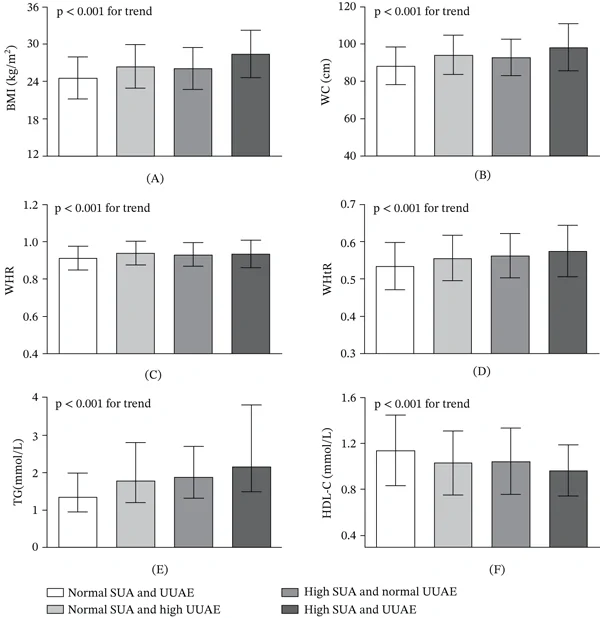
Comparisons of parameters related to obesity and MetS among the four groups. (A) Comparison of BMI levels among Group 1, Group 2, Group 3, and Group 4 after adjusting for gender, age, and DD (*p* < 0.001 for trend). (B) Comparison of WC levels among Group 1, Group 2, Group 3, and Group 4 after adjusting for gender, age, and DD (*p* < 0.001 for trend). (C) Comparison of WHR levels among Group 1, Group 2, Group 3, and Group 4 after adjusting for gender, age, and DD (*p* < 0.001 for trend). (D) Comparison of WHtR levels among Group 1, Group 2, Group 3, and Group 4 after adjusting for gender, age, and DD (*p* < 0.001 for trend). (E) Comparison of TG levels among Group 1, Group 2, Group 3, and Group 4 after adjusting for gender, age, and DD (*p* < 0.001 for trend). (F) Comparison of HDL‐C levels among Group 1, Group 2, Group 3, and Group 4 after adjusting for gender, age, and DD (*p* < 0.001 for trend).

### 3.5. Associations of SUA and UUAE Groupings With Obesity and MetS in T2DM

Table 2 presents the associations between different groups and the presence of obesity, abdominal obesity, and MetS in T2DM. The odds of obesity, abdominal obesity, and MetS were significantly higher in Groups 2 and 3 than in Group 1 even after full adjustment for confounders (Group 2, OR: 1.524, 95% CI: 1.185–1.960 for obesity; OR: 1.652, 95% CI: 1.245–2.192 for abdominal obesity; and OR: 1.440, 95% CI: 1.057–1.962 for MetS; Group 3, OR: 1.494, 95% CI: 1.284–1.739 for obesity; OR: 1.622, 95% CI: 1.343–1.960 for abdominal obesity; and OR: 2.526, 95% CI: 1.991–3.206 for MetS, *p* < 0.001 for all trends). Furthermore, compared with Groups 2 and 3, Group 4 had higher odds of obesity and abdominal obesity (OR: 4.160, 95% CI: 2.102–8.234 for obesity and OR: 5.405, 95% CI: 2.115–13.811 for abdominal obesity, *p* < 0.001 for both trends). Notably, in the fully adjusted model (Model 6), Group 4 still exhibited the highest odds of MetS (OR: 3.140, 95% CI: 1.218–8.095 for MetS, *p* < 0.001 for trend).

**Table 2:** The association of SUA and UUAE groupings with obesity and MetS in T2DM.

	ORs (95% CI)				*p* values for trend
	Patients with normal SUA and UUAE	Patients with normal SUA and high UUAE	Patients with high SUA and normal UUAE	Patients with high SUA and UUAE	
*Obesity*					
Model 1	1	2.320 (1.879–2.863)	2.256 (1.987–2.561)	7.413 (3.998–13.745)	< 0.001
Model 2	1	2.202 (1.782–2.722)	2.301 (2.025–2.613)	6.820 (3.672–12.666)	< 0.001
Model 3	1	2.206 (1.785–2.728)	2.297 (2.021–2.610)	7.422 (3.905–14.107)	< 0.001
Model 4	1	2.121 (1.709–2.632)	2.297 (2.016–2.617)	7.845 (4.101–15.005)	< 0.001
Model 5	1	2.015 (1.619–2.506)	2.218 (1.943–2.531)	7.865 (4.087–15.134)	< 0.001
Model 6	1	1.524 (1.185–1.960)	1.494 (1.284–1.739)	4.160 (2.102–8.234)	< 0.001
*Abdominal obesity*					
Model 1	1	1.958 (1.544–2.484)	2.616 (2.245–3.048)	8.784 (3.544–21.772)	< 0.001
Model 2	1	2.514 (1.969–3.209)	2.556 (2.184–2.991)	11.596 (4.647–28.941)	< 0.001
Model 3	1	2.460 (1.926–3.141)	2.557 (2.184–2.993)	11.496 (4.602–28.721)	< 0.001
Model 4	1	2.306 (1.799–2.957)	2.561 (2.182–3.005)	11.858 (4.723–29.774)	< 0.001
Model 5	1	2.232 (1.738–2.866)	2.449 (2.084–2.878)	11.832 (4.698–29.799)	< 0.001
Model 6	1	1.652 (1.245–2.192)	1.622 (1.343–1.960)	5.405 (2.115–13.811)	< 0.001
*MetS*					
Model 1	1	1.974 (1.520–2.563)	4.304 (3.525–5.254)	5.293 (2.294–12.215)	< 0.001
Model 2	1	2.358 (1.809–3.075)	4.170 (3.410–5.098)	6.465 (2.787–15.000)	< 0.001
Model 3	1	2.292 (1.757–2.990)	4.262 (3.479–5.220)	6.490 (2.792–15.082)	< 0.001
Model 4	1	2.143 (1.637–2.805)	4.286 (3.493–5.259)	6.386 (2.735–14.914)	< 0.001
Model 5	1	2.022 (1.539–2.657)	3.997 (3.249–4.918)	6.209 (2.642–14.594)	< 0.001
Model 6	1	1.440 (1.057–1.962)	2.526 (1.991–3.206)	3.140 (1.218–8.095)	< 0.001

*Note:* Model 1: unadjusted. Model 2: adjusted for age, gender, and DD. Model 3: additionally adjusted for hypertension, smoking, and alcohol drinking. Model 4: additionally adjusted for the use of IIAs, metformin, and SUs. Model 5: additionally adjusted for SBP and DBP. Model 6: further adjusted for ALT, TC, HDL‐C, LDL‐C, TG, eGFR, Cr, UAE, FPG, 2‐h PPG, HbA1c, FCP, 2‐h C‐P, and CRP.

### 3.6. Comparisons of CRP and HOMA2‐IR Levels in Different Groups

Figure 4 displays the comparison of CRP and HOMA2‐IR levels in different groups. Group 4 showed the highest CRP levels, whereas CRP levels were also significantly higher in Groups 2 and 3 compared to Group 1 (Figure 4A, *p* < 0.001 for trend). In addition, CRP levels markedly increased with the severity of obesity and abdominal obesity (Figure 4B,C, *p* < 0.001 for both trends). Furthermore, CRP levels also showed a significant increase with the accumulation of the number of MetS components (Figure 4D, *p* < 0.001 for trend). Consistent with the findings for CRP, HOMA2‐IR levels were also significantly higher in Groups 2 and 3 compared to Group 1, with the highest level in Group 4 (Figure 4E, *p* < 0.001 for trend). With the severity of obesity and abdominal obesity and the increase in combined MetS components, HOMA2‐IR levels significantly increased (Figure 4F–H, *p* < 0.001 for all trends).

**Figure 4:**
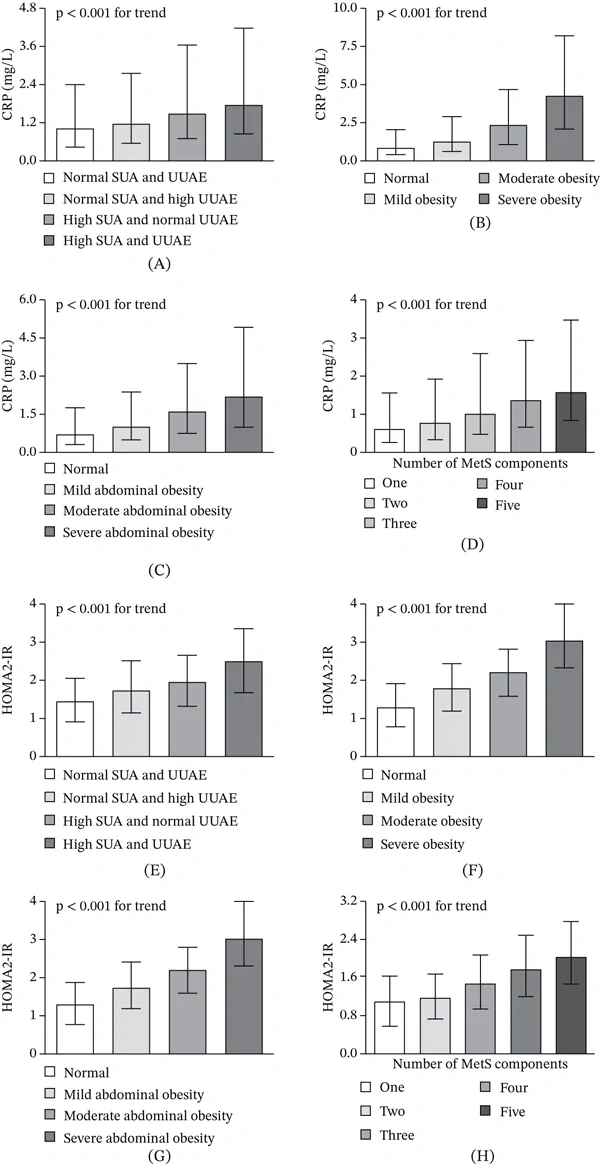
Comparisons of CRP and HOMA2‐IR levels among different groups. (A) Comparison of serum CRP levels among Group 1, Group 2, Group 3, and Group 4 after controlling for gender, age, and DD (*p* < 0.001 for trend). (B) Comparison of serum CRP levels stratified by normal, mild obesity, moderate obesity, and severe obesity after controlling for gender, age, and DD (*p* < 0.001 for trend). (C) Comparison of serum CRP levels stratified by normal, mild abdominal obesity, moderate abdominal obesity, and severe abdominal obesity after controlling for gender, age, and DD (*p* < 0.001 for trend). (D) Comparison of serum CRP levels stratified by the number of MetS components after controlling for gender, age, and DD (*p* < 0.001 for trend). (E) Comparison of serum HOMA2‐IR levels among Group 1, Group 2, Group 3, and Group 4 after controlling for gender, age, and DD (*p* < 0.001 for trend). (F) Comparison of serum HOMA2‐IR levels stratified by normal, mild obesity, moderate obesity, and severe obesity after controlling for gender, age, and DD (*p* < 0.001 for trend). (G) Comparison of serum HOMA2‐IR levels stratified by normal, mild abdominal obesity, moderate abdominal obesity, and severe abdominal obesity after controlling for gender, age, and DD (*p* < 0.001 for trend). (H) Comparison of serum HOMA2‐IR levels stratified by the number of MetS components after controlling for gender, age, and DD (*p* < 0.001 for trend).

## 4. Discussion

This cross‐sectional, real‐world study showed that both high SUA and high UUAE levels were significantly associated with the increased prevalence of obesity and MetS in T2DM subjects. More importantly, the coexistence of hyperuricemia and high UUAE was associated with even higher odds of obesity, abdominal obesity, and especially MetS, even after adjustment for multiple confounding factors. These findings underscore the important role of UA in various metabolic diseases in individuals with T2DM.

In the present study, the prevalence of obesity in T2DM patients was 48.6% in men and 46.7% in women, without a significant difference between genders. However, the prevalences of abdominal obesity and MetS were 81.0% and 79.4%, respectively, in women, significantly higher than the corresponding prevalences of 56.6% and 65.0% in men. Similar to the present study, Ezenwaka et al. found that the prevalence of obesity in T2DM patients was similar between genders, but the prevalence of abdominal obesity was remarkably higher in female patients (97.7%) compared with male patients (68.9%) [23]. In addition, one study also found that the prevalence of MetS among individuals with T2DM was significantly higher in females (67.0%) compared with males (40.0%) [24]. Furthermore, the prevalence of abdominal obesity and MetS showed a rising trend with age and DD, whereas there was no obvious trend in obesity prevalence. Therefore, the prevalence of abdominal obesity and MetS appeared to be relatively higher among female, older, and long‐duration T2DM patients.

Currently, a variety of studies have also observed a clearly positive correlation between SUA and obesity in the general population [18, 25, 26]. For example, consistent with the present findings, a significant increase in the prevalence of obesity was found across SUA quartiles in a general population, and both SUA quartiles and levels were obviously correlated with BMI and WC [25]. Additionally, a significantly positive association between hyperuricemia and obesity was also observed in the general population in both genders even after multiple adjustments (male OR = 3.165, *p* < 0.001; female OR = 3.776, *p* < 0.001) [26]. In addition, many studies have demonstrated a significant association between SUA levels and multiple metabolic diseases such as MAFLD and carotid atherosclerosis [12–14]. In line with the findings of the present study, a nationwide cross‐sectional study also reported a significant positive association between elevated SUA and MetS in general patients (OR = 2.608, 95% CI: 2.281–2.982) [27].

However, few studies have investigated the relationship between SUA levels and obesity and MetS in patients with T2DM. In line with the present study, Zong et al. noted that in T2DM subjects, SUA levels were not only significantly associated with BMI and WC but also exhibited a strong correlation with central adiposity, including visceral adipose tissue (VAT), subcutaneous adipose tissue (SAT), android fat mass, and gynoid fat mass [28]. Likewise, recent studies also supported a strong positive association between SUA and obesity and MetS in the Chinese population with T2DM [15, 16]. Consistent with the findings mentioned above, the present study also showed that T2DM patients with elevated SUA exhibited a higher prevalence of obesity, abdominal obesity, and MetS compared to subjects with normal SUA.

However, unlike SUA, studies on the association between UUAE and metabolic dysfunctions are sparsely available, and the findings are inconsistent. Recently, a remarkably positive correlation was reported between UUAE and NAFLD in T2DM [29]. In contrast, also in the T2DM population, previous studies revealed that low levels of UUAE were an independent risk factor for CKD as well as diabetic retinopathy [30, 31]. A study of 200 Chinese patients with CKD observed that BMI increased statistically across tertiles of 24‐h UUAE [20]. Likewise, the present study found a significantly positive correlation between the prevalence of obesity, abdominal obesity, and MetS and UUAE levels in T2DM patients. In contrast, Li et al. found that there was no significant relationship between 24‐h UUAE tertiles and BMI in 428 adult patients with CKD (*p* = 0.061) [32].

More importantly, the present study demonstrated that the coexistence of high SUA and high UUAE levels was associated with even higher odds of obesity, abdominal obesity, and MetS in T2DM. Currently, there is a lack of studies investigating the association of the combined presence of hyperuricemia and high UUAE with obesity and MetS in T2DM patients. Given that SUA and UUAE measurements are simple and noninvasive, their combined assessment may help identify patients with T2DM who have a higher likelihood of obesity, abdominal obesity, or MetS.

Furthermore, the results showed that obesity‐related indicators, including BMI, WC, WHR, and WHtR, were significantly higher in T2DM patients with either hyperuricemia or high UUAE compared with those with normal SUA and UUAE. In addition, the MetS‐related parameter TG showed the same trend, whereas HDL‐C exhibited an opposite trend. Additionally, more deterioration in obesity and MetS parameters was found in the patients with combined hyperuricemia and high UUAE. Moreover, the highest observed prevalence of severe obesity, abdominal obesity, and MetS was found in patients with both high SUA and high UUAE. In agreement with these findings, a significant correlation between SUA and BMI as well as WHtR was noted in a study including 2248 overweight and obese children and adolescents [33]. In addition, a 10‐year follow‐up cohort study found that elevated SUA was significantly associated with the incidence of dyslipidemia, showing a positive correlation with TG and a negative correlation with HDL‐C [34]. Moreover, in idiopathic calcium oxalate stone formers, Siener et al. demonstrated that both serum and urinary UA levels increased with increasing severity of obesity [35]. Similarly, higher UUAE was found to be significantly associated with more severe dyslipidemia characterized by high TG and low HDL‐C [36]. Thus, the coexistence of high levels of SUA and UUAE significantly augments the severity of obesity, abdominal obesity, and MetS.

Finally, the present study showed a significant increase in CRP and HOMA2‐IR levels in patients with either elevated SUA or UUAE compared with those with normal SUA and UUAE and a further increase in patients with combined high SUA and UUAE. In addition, serum CRP and HOMA2‐IR levels were positively correlated with obesity, abdominal obesity, and MetS. These findings are compatible with the possible involvement of inflammation and IR. However, these mechanisms were not directly assessed, and no causal pathway can be inferred from the present study. It is generally accepted that obesity and MetS are characterized by the presence of systemic chronic low‐grade inflammation [37, 38] and that CRP is regarded as a reliable indicator of inflammation, which is elevated in chronic inflammatory diseases [39]. Clinically, similar to the present study, Mazidi et al. reported a significant increase in serum CRP levels with increasing SUA quartiles [40]. Therefore, inflammation might partially account for the observed association between elevated SUA and UUAE and increased severity of obesity and MetS. In addition, as typical metabolic disorders, both obesity and MetS share insulin resistance as a key pathological mechanism [41]. HOMA2‐IR, a commonly used clinical index of IR, has been demonstrated to be positively associated with SUA levels [42]. These findings suggested that IR may also play a critical role in mediating the relationship between elevated SUA and UUAE and the exacerbation of obesity and MetS. Notably, approximately 70% of the UA pool is excreted through the kidney, of which 90% is reabsorbed in the proximal renal tubules, with the remaining 10% ultimately excreted in the urine [43]. Therefore, elevated SUA may lead to increased UUAE when the reabsorption threshold is exceeded. Therefore, the coexistence of hyperuricemia and elevated UUAE suggested higher levels of UA in vivo. Consequently, the coexistence of high SUA and UUAE may be associated with even higher odds and greater severity of obesity and MetS, which may be related to the proinflammatory and IR effects of UA. However, neither pathway was evaluated as a causal mediator in this cross‐sectional study, and these interpretations require confirmation in longitudinal studies and mechanistic experiments.

The novelty of this study lies in jointly evaluating SUA and UUAE in relation to obesity, abdominal obesity, and MetS in patients with T2DM, extending previous work that examined either measure separately. Our findings show that coexisting hyperuricemia and high UUAE are associated with higher odds of these metabolic conditions, providing additional evidence on the metabolic profile of patients with both abnormalities.

## 5. Limitations and Recommendations for Future Research

However, several limitations should be acknowledged. Firstly, the present study was a cross‐sectional study, which limited the ability to establish a causal relationship between elevated SUA and/or UUAE and the development of obesity and MetS. Second, as the present study was conducted at a single center, future prospective multicenter studies are needed to validate these findings. The hospital‐specific UUAE cutoff may also limit comparability across laboratories and requires external validation. In addition, the relatively small sample size of Group 4 may have reduced the precision of the estimates, and the results for this group should therefore be interpreted with caution. Furthermore, the absence of detailed dietary intake and physical activity data may have introduced residual confounding, and future studies should incorporate these factors. Finally, the study population comprised patients with T2DM, so the present findings may not be applicable to other populations. Future studies are needed to validate these associations in a wider range of populations and explore potential biological mechanisms.

## 6. Conclusion

In conclusion, hyperuricemia and elevated UUAE were independently associated with obesity and MetS among patients with T2DM, and their coexistence was associated with higher odds of both conditions. In clinical practice, the combined determination of SUA and UUAE levels may help to identify patients with T2DM who have a greater burden of metabolic abnormalities.

## Author Contributions

Lian‐Xi Li and Zhi‐Feng Sheng: conceptualization, resources, project administration, supervision, funding acquisition, and investigation. Man‐Rong Xu, Meng‐Han Li, and Cui‐Chun Zhao: data curation, formal analysis, software, visualization, and writing—original draft. Lian‐Xi Li, Yu‐Jie Wang, Ming‐Yun Chen, Ya‐Wen Zhang, Jun‐Xi Lu, Chun Yue, and Lu‐Lu Xu: methodology, validation, and writing—review and editing. Man‐Rong Xu, Meng‐Han Li, and Cui‐Chun Zhao are cofirst authors.

## Funding

This study was funded by the Noncommunicable Chronic Diseases‐National Science and Technology Major Project (2026ZD0558000 and 2026ZD0558005).

## Disclosure

All authors contributed to the article and approved the submitted version.

## Ethics Statement

This study obtained written informed consent from all subjects and was approved by the Ethics Committee of the Shanghai Sixth People′s Hospital affiliated to Shanghai Jiao Tong University School of Medicine (Approval Number: 2018‐KY‐018(K)).

## Conflicts of Interest

The authors declare no conflicts of interest.

## Data Availability

The data that support the findings of this study are available from the corresponding authors upon reasonable request.
